# Bidirectional reinforcement learning neural network for constrained molecular design

**DOI:** 10.1038/s41598-025-33443-3

**Published:** 2025-12-24

**Authors:** Junan Lin, Jiří Hostaš, Anguang Hu, Hang Hu, Hsu Kiang Ooi, Mohammad Sajjad Ghaemi

**Affiliations:** 1 Digital Technologies Research Centre, National Research Council Canada, Toronto, ON Canada; 2 Suffield Research Centre, Defence Research and Development Canada, Medicine Hat, AB Canada

**Keywords:** Chemistry, Computational biology and bioinformatics, Drug discovery, Mathematics and computing

## Abstract

We present BiRLNN, a bidirectional molecular design framework that combines recurrent neural networks with reinforcement learning to optimize drug-like properties of generated compounds. We examined the use of Self-Referencing Embedded Strings representations, which ensures 100% syntactic validity of generated molecules. By generating molecular sequences in both forward and backward directions, we enabled more balanced exploration of chemical space while maintaining constraint requirements during molecular design. To guide generation towards desirable pharmacological targets, we implement a multi-objective reward function based on quantitative estimate of drug-likeness and synthetic accessibility, and apply policy gradient-based reinforcement learning for fine-tuning. We demonstrate that our bidirectional model covers the full constrained chemical space compared to unidirectional ones using pharmaceutically relevant fragments, allowing it to explore regions containing molecules unreachable by the latter. Moreover, the reinforcement learning process successfully steers the constrained generation process toward desirable compound classes with improved reward metrics. Our results demonstrate that BiRLNN offers a robust and flexible strategy for navigating chemical space in multi-objective drug design tasks.

## Introduction

Drug discovery is a challenging and resource-intensive endeavor due to the combinatorial search space of chemical domain, which has been estimated to span from $10^{23}$ to $10^{60}$ candidate molecules^1^. Developing a new drug often requires over a decade of research and can cost as much as 2.8 billion USD^2^. To address this bottleneck, recent advances in artificial intelligence (AI), particularly deep learning, have shown significant promise in accelerating molecular discovery by generating novel structures with desired pharmacological properties.

Recurrent neural networks (RNNs) have been widely adopted for molecular design tasks due to their ability to model sequential data effectively^3^. In particular, a variant of RNN based on long short-term memory (LSTM) cells^4^ is frequently used instead of the original RNN due to its ability to overcome the exploding/diminishing gradient problem. In this work, the term RNN shall always be understood as referring to the LSTM variant, unless stated otherwise. A major limitation of traditional unidirectional RNNs is their left-to-right generation process, which may fail to fully capture structural information, especially when constraints are present. Bidirectional RNNs^5,6^ provide a solution to overcome this limitation by processing sequences and capturing long-range dependencies in both forward and backward directions. This capability is especially advantageous in constrained molecular design tasks, where specific structures must be preserved in the generated string. While unconstrained generation has been explored generically^7^ and towards specific targets such as central nervous system drugs^8^, constrained generation remains a less explored area. Constrained molecular design can be a useful strategy for several reasons: first, it allows the generated candidates to automatically retain known functional groups or pharmacophores that are known to be useful for specific tasks. Specifically, prior knowledge about structure-activity relationships can guide the exploration of chemical space more effectively. While an unconstrained scheme can also produce target-containing structures, the vast size of the chemical space imply that constrained design can be much more efficient in producing meaningful candidates. Second, constraints can be used to improve synthetic accessibility by including substructures known to be synthetically feasible, reducing potential costs in the production stage. Finally, constrained generation facilitates the optimization of molecular scaffolds, enabling the design of analogues around a lead compound while preserving core chemical frameworks. This approach improves the relevance and hit-rate of generated molecules, making the overall design process more efficient and interpretable. Noticeable efforts along this direction include the SMILES-based scaffold decorator method^9^ and molecular graph-based methods^10,11^.

While bidirectional models offer advantages in constrained design, another challenge comes from property-based optimization, where it is often desirable for drug lead molecules to satisfy multiple (and sometimes conflicting) criteria, including but not limited to binding affinity, toxicity, synthetic accessibility, and molecular weight. These requirements make molecule generation a multi-parameter optimization (MPO) task. Reinforcement learning (RL) provides a powerful framework to address MPO problems through reward-based optimization. Early successes in this area include the DeepFMPO framework^12^which combined a LSTM model with an actor-critic method to improve lead molecules via fragment-based molecular design, using a constraint-based reward system: if a particular property of a generated molecule falls within the desired range of value, it contributes a value of 1 towards the total reward, and 0 otherwise. Zhavoronkov et al^13^. introduced GENTRL which combined autoencoder architecture with a composite reward function defined using self-organizing maps to design kinase inhibitors. Popova et al^14^. proposed ReLeaSE that combines stack-augmented RNNs with gated recurrent unit (GRU) cells with RL to produce chemical libraries with desired properties. Their approach was later used by Goel et al^15^. with an alternating reward, and by Hu et al^16^. with a memory storage network, both for the purpose of increasing the diversity of the generated molecules. Segler et al^17^. employed bidirectional LSTMs for retrosynthetic pathway prediction, illustrating their capability to model chemical transformations bidirectionally. Li et al^18^. proposed a forward-backward generation approach that alternates between left-to-right and right-to-left synthesis to improve structural coherence and validity. Gómez-Bombarelli et al^19^. introduced variational autoencoders (VAEs) for generating novel molecules by encoding Simplified Molecular Input Line Entry System (SMILES) strings into a continuous latent space. Additionally, $\text {CHA}_2$ successfully leverages the autoencoder’s latent space, which is constrained within a convex hull, to restrict the region of interest and generate out-of-distribution molecular candidates^20^. Despite these innovations, these models produce molecular designs in an unconstrained manner, limiting their ability to preserve known functional groups or structures.

Another important link between sequential learning and molecular design is the encoding of molecular structures into strings, which can be understood by the machine learning models. A popular approach is to utilize string-based encoding schemes, such as the SMILES^21^ or Self-Referencing Embedded Strings (SELFIES)^22^. These encodings offer a convenient and interpretable format for AI-based molecular design, since they can be interpreted in a way similar to conventional text data by the RNNs. Both representations provide a linearization of molecular graphs, making them well-suited for sequential learning. Among these, SELFIES provides a key advantage: its internal encoding guarantees that every sequence corresponds to a valid molecule, making it highly robust to arbitrary combinations^23^. Despite this robustness, SMILES has dominated over SELFIES for historical reasons, where the latter only started to gain attention in AI-based molecular design in more recent studies^24^.

Other recent approaches to molecular design have moved beyond RL, adopting alternative optimization strategies. Notable examples include the GFlowNet^25^ that implements multi-objective bayesian optimization by Zhu et al., FFLOM^26^ which utilizes a flow-based autoregressive model by Jin et al., MARS^27^ which is a fragment-based Markov sampling algorithm by Xie et al., DecompDiff^28^ which utilizes a diffusion model with decomposed ligand priors by Guan et al., GEAM^29^ which combines fragment extraction, assembly, and modification in a goal-aware manner by Lee et al., VNFlow^30^ which combines VAE with normalizing flow to produce high quality organophosphate molecules by Hostaš et al., just to name a few. While these methods offer compelling alternatives, they often require complex architectures or are not easily adaptable to constrained generation.

In this work, we propose the Bidirectional Reinforcement Learning Neural Network (BiRLNN) framework for constrained molecular design. Our method builds upon existing LSTM-based models including FBRNN and BIMODAL^7^, while exploring both SMILES and SELFIES representations, where the latter guarantees validity of generated molecules. This bidirectional generation scheme improves structural variations, and expands the search domain in chemical space. We comprehensively benchmark the generation quality starting from six pharmaceutically relevant initial substrings, demonstrating how initial constraints modify the shape of chemical spaces as well as the resulting molecular properties. To further tailor molecules toward target properties, BiRLNN integrates a RL loop using a modified REINFORCE^31^ algorithm. Our method aligns with the broader goal of incorporating active learning into generative molecular design, an idea that traces back decades^32^ but has recently been revitalized by advances in deep learning^33,34^. By integrating bidirectional modeling, robust SELFIES encoding, and RL under a unified architecture, BiRLNN offers a flexible and interpretable solution to the problem of constrained generation and optimization of drug-like molecules.

In the following sections, we detail the BiRLNN architecture, training procedures, and experimental validations. We demonstrate its ability to generate diverse, chemically valid molecules that meet multiple design objectives, thereby offering a flexible and efficient framework for generating chemically valid and pharmaceutically relevant molecules.

## Model architecture and design

Our framework builds upon and extends the concept of bidirectional generation, inspired by the asynchronous forward/backward model introduced by Mou et al^35^. and further explored under the name BIMODAL by Grisoni et al.^7^. Meanwhile, we added two key modifications for improvements. First, in addition to the SMILES molecular string representation used in^7^, we explored molecular encoding in SELFIES strings^23^ and showed its advantages as an encoding scheme for string-based molecular generation tasks using RNN architecture. Moreover, we demonstrated the advantage of using a bidirectional scheme in a constrained generation task compared to uni-directional ones by illustrating the sizes of spaces covered by both models, further strengthening the importance of applying bidirectional schemes. Second, we added an RL loop which fine-tunes the model weights according to a reward function, such that it is more likely to produce molecules with a high reward. This enables a targeted fine-tuning process that is not present in^7^, allowing score-guided designs for better drug candidates.

### RNNs with LSTM cells

**Fig. 1:**
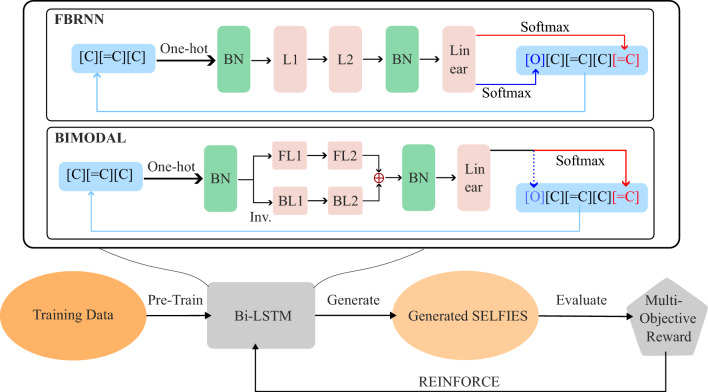
BiRLNN framework. The internal structure of the two bidirectional models are visualized, as well as their generation schemes. The abbreviations (BN, L1/2, FL1/2, BL1/2) stand for (BatchNormalization, LSTM1/2, Forward-LSTM1/2, BackwardLSTM1/2). “Inv” means inverting the input string for the BackwardLSTM. After initial training using a drug molecule dataset, the model enters a fine-tuning stage where each epoch consists of generating a batch of SELFIES strings, and the parameters of Bi-LSTM are updated using the batch REINFORCE algorithm, with the batch average award as the bias.

In this work we work with RNNs with LSTM cells, which were designed to solve the vanishing/exploding gradient issue present in the original RNN architecture^4^. RNNs fulfill the generation task by predicting the next token(s) using the existing molecular string and extends it, and the process repeats until certain ending criteria (typically either when a special end-of-string token is sampled or when the maximum length is reached) are fulfilled. At time step *t*, given an input $x_t$, the LSTM model uses 3 different sets of weights and biases to compute the input $i_t$, forget $f_t$, and output $o_t$ gates, as well as the new hidden state $h_t$, via the formula

1 $$\begin{aligned} \begin{aligned} i_t&= \sigma (W_i x_t + U_i h_{t-1} + b_i)\\ f_t&= \sigma (W_f x_t + U_f h_{t-1} + b_f)\\ o_t&= \sigma (W_o x_t + U_o h_{t-1} + b_o)\\ \tilde{c}_t&= \tanh {(W_c x_t + U_c h_{t-1} + b_c)}\\ c_t&= f_t \circ c_{t-1} + i_t \circ \tilde{c}_{t}\\ h_t&= o_t \circ \tanh {(c_t)} \end{aligned} \end{aligned}$$

where *W* and *b* are the sets of weights and biases, $\sigma$ and $\circ$ denote the sigmoid function and Hadamard (element-wise) product respectively. To generate a final prediction for the output token, the hidden state is processed differently depending on the variant of the LSTM network used. In this work we examined the following 3 variants:

- *Forward-RNN and Backward-RNN*. The forward-RNN model consists of 5 layers (BatchNormalization 1, LSTM 1, LSTM 2, BatchNormalization 2, Linear). In this model, information is processed uni-directionally from left to right. The output from the *t*-th time step is simply the hidden state value, $y_t = h_t$. Given an initial string $\textbf{x}(i)$ at time step *t*, forward-RNN predicts the probability of next token to the right via the softmax function over the output logit vector $\textbf{y}_t$ with temperature parameter *T*:
  2 $$\begin{aligned} P(x_{t+1}=k | \textbf{x}_t) = \frac{\exp (y_{t, n}/T)}{\sum _{n=1}^{K} \exp (y_{t, n}/T)}. \end{aligned}$$
  Then, a new token is sampled following this distribution, and is appended to the right of $\textbf{x}_t$ to form $\textbf{x}_{t+1}$. By the same principle, a Backward-RNN model receives the training token in reversed order, predicts the probability of the previous token given the current inverted string, and append the sampled token to its left to form the new string at time $t+1$.
- *FB-RNN*. The FB-RNN model^35^ has the same 5 layers as the forward-RNN model. It predicts both the next and previous token given an input string $\textbf{x}(i)$ at time step *t*, by producing two independent conditional probability vectors $\textbf{y}_{t+}$ and $\textbf{y}_{t-}$. This is done by doubling the dimension of both the input to the BatchNormalization 1 layer, and the output from Linear layer. The new forward and backward tokens are produced via the same softmax function over $\textbf{y}_{t+}$ and $\textbf{y}_{t-}$ as in section "RNNs with LSTM cells", where $\textbf{y}_{t+}$ and $\textbf{y}_{t-}$ are the first and second halves of the Linear layer output. Thus, the FB-RNN model generates two tokens per iteration towards both ends, as shown in Fig. 1.
- *BIMODAL*. The BIMODAL model^7^ consists of 7 layers (BatchNormalization 1, F-LSTM 1, B-LSTM 1, F-LSTM 2, B-LSTM 2, BatchNormalization 2, Linear). BIMODAL reads the current string along both the forward and backward directions for an input string at time step *t*, using two separate RNNs (one for each direction). The output from BIMODAL is given by
  3 $$\begin{aligned} y_t = W_{hy, +} h_{t, +} +W_{hy, -} h_{t, -} +b_{hy} \end{aligned}$$
  where $W_{hy, \pm }$ are the hidden-to-output weight matrix for forward/backward prediction, $h_{t, \pm }$ are the hidden states, and $b_{hy}$ is the bias vector. In our implementation, this is achieved by enabling the bidirectional=True option for the torch.nn.LSTM class in PyTorch^36^. The actual generation is then done in an alternating manner, producing a new token in the forward direction for odd steps or in the backward direction for even steps. This is represented by the solid and dashed lines at the generation step in Fig. 1.

### Reinforcement learning

The next component in the BiRLNN network is the refinement of the Bi-LSTM model using RL. A typical setting of an RL task involves an agent interacting with an environment, observing its state $s_t$ at each step *t* while making an action $a_t$. The important components include: (1) a policy parametrized by $\theta$ that maps states to a probability distribution over actions $a_t$; (2) a reward function $R_t$ that assigns scalar feedback to actions taken in an environment; (3) a trajectory $\tau$, which is a sequence of state-action-reward transitions; and (4) an objective function, usually the expected return, which the algorithm aims to maximize. Here, our discussion focuses on one realization of RL using the REINFORCE algorithm^31^, which belongs to the family of Monte Carlo policy gradient methods. While more advanced policy gradient methods such as Proximal Policy Optimization (PPO)^37^ and Trust Region Policy Optimization (TRPO)^38^ exist, they are primarily designed for continuous action spaces and often require extensive hyperparameter tuning and large batch sizes to achieve stable performance. In the context of molecular design, where actions correspond to discrete token selections and the reward signals are sparse and non-differentiable, these algorithms tend to offer limited practical benefit, although recent efforts started to show evidence for their potential advantage^39^. Prior works in this domain^40–42^ has confirmed the effectiveness of REINFORCE in the task of optimizing discrete molecular sequences.

In REINFORCE, the policy parameters are updated stochastically using the gradient of the log-probability of the actions taken, scaled by the return received. Mathematically, this can be written as $\mathbb {E}_\tau [\sum _{t} \nabla _\theta \log \pi _\theta (a_t \mid s_t) R_t]$. While simple and broadly applicable, REINFORCE in its basic form suffers from high variance and slow convergence, motivating various variance reduction techniques. Here, we augment the basic REINFORCE with several improvements to make it more robust against aforementioned problems. First, we apply baseline subtraction, where an arbitrary baseline function $b(s_t)$ from the raw reward function $R_t$. It is known that as long as *b* only depends on the state $s_t$ and not the action, the resulting policy gradient estimator remains unbiased, but could have a lower variance for properly chosen *b*^43^. Next, batching multiple trajectories together further smooths gradient estimates and helps mitigate the stochasticity inherent in individual episodes. Finally, we include an additional entropy bonus term, which measures the level of concentration during each rollout:

4 $$\begin{aligned} H(\pi _\theta (\cdot |s)) = -\sum _{a} \pi _\theta (a \mid s) \log (\pi _\theta (a \mid s)) \end{aligned}$$

to encourage the model to further explore rather than settling on premature solutions. With these methods integrated, we can write down the policy gradient update rule used in this work as:

5 $$\begin{aligned} \theta ' = \theta + \alpha \left( \frac{1}{K} \sum _{i=1}^{K} \sum _{t=0}^{T_i} \nabla _\theta \log \pi _\theta (a_t^i \mid s_t^i) (R_t^i - b(s_t^i)) \right) + \beta \left( \nabla _\theta \frac{1}{K} \sum _{i=1}^{K} \sum _{t=0}^{T_i}H(\pi _\theta (\cdot \mid s_t^i)) \right) , \end{aligned}$$

where $\theta$ are the policy parameters, $\alpha$ is the learning rate, $\beta$ is the entropy coefficient, and *K* is the batch size.

For BiRLNN model in molecular generation, the policy corresponds to the LSTM models responsible for producing molecular strings sequentially. Each trajectory (or rollout) consists of the production of a full molecular string, starting from an initial string. The reward function is constructed as a linear combination of rescaled quality measures of the completed molecule:

6 $$\begin{aligned} R_t(\textbf{p}) = \sum _l w_{t,l} p_{t,l} \end{aligned}$$

where $\textbf{p}$ denotes the set of metrics that we wish to optimize in the RL process, and $w_l$ are the set of weights denoting their relative importance. Since these metrics can only be evaluated at the end of a generation process, one may assign $p_{t,l}=0$ for $t=0, 1, \dots , T-1$ since they cannot be evaluated for an incomplete string, causing the reward trajectory to also be sparse. This sparsity leads to a simplification in the policy gradient update rule, since we now only need to sum over all the sampled molecules in a batch. Moreover, this provides a simple expression for *b*(*s*): since it is desirable to use the on-policy value as the baseline, a simple non-biased estimator is just the average of final rewards from each episode in the batch $\bar{R}$. This leads to the final expression for the policy parameter update rule,

7 $$\begin{aligned} \theta ' = \theta + \alpha \left( \frac{1}{K} \sum _{i=1}^{K} \nabla _\theta \log \pi _\theta (a^i \mid s^i) (R^i - \bar{R}) \right) + \beta \left( \nabla _\theta \frac{1}{K} \sum _{i=1}^{K} \sum _{t=0}^{T_i}H(\pi _\theta (\cdot \mid s_t^i)) \right) , \end{aligned}$$

which is repeated for multiple episodes until the batch average reward $\bar{R}$ is no longer improving. The reward function for the RL task can, in general, include multiple aspects about the actions taken by the agent. These can be combined into a single multi-objective reward signal, which drives the policy gradient updates.

## Results

### Building and training the Bi-LSTM models

We perform a comprehensive benchmark analysis on our SELFIES-based bidirectional model against the previous SMILES-based ones by Grisoni et al.^7^. In selecting the generation methods, we skipped the NADE method which was found to have a suboptimal performance for molecular design. Also, we skipped the data augmentation which was found to have only a small effect on the generation quality, while significantly increasing the training overhead. Meanwhile, we kept the the position of the initial dummy token by either placing it at the middle of the string (fixed), or randomly place it within the string (random) to test its. We reused the optimal hyperparameters obtained in the previous SMILES model trainings, including model architectures, model sizes, and learning rates. For consistency, the same set of 271,914 molecules was used as the raw training dataset, which has been filtered from the CHEMBL dataset (version 22)^44^. We translated all the SMILES strings in the original training set into SELFIES strings, and performed one-hot encoding to obtain the SELFIES training set. The actual training set is then formed by inserting a dummy initial token (“G” for SMILES strings and “[G]” for SELFIES strings) into the raw strings, and aligning the lengths of all strings by using a padding token (“A” or “[A]”). All molecular strings are padded to the same length of the longest encoded string for its category: 76 tokens for SMILES strings, and 83 tokens for SELFIES strings. Each model is trained for 10 epochs (each epoch being a pass of the full training set), using a 5-fold cross-validation (CV) scheme. Each trained model is then used to sample 100 molecular strings, where the model outputs the full-length strings for every generation, including the initial and padding tokens which are removed by a post-processing procedure. we compute the following statistics among the 5-fold CV runs:

1. % Unique: percentage of molecular strings (SMILES/SELFIES) that are not repetitive in the generated set.
2. % Valid: percentage of unique molecular strings that are syntactically valid.
3. % Novel: percentage of valid molecular strings that are not present in the training set.

Next, we sampled a larger set of 6, 000 molecular strings using the trained model from each CV fold, constraining them to be unique, valid, and novel by discarding generated strings that do not satisfy the constrains, leading to a total of 30, 000 molecules. We then compute the Fréchet ChemNet Distance^45^ (FCD) between each generated set and the training set. The FCD is a measure of similarity between two molecular distributions, in the latent space of the ChemNet model which predicts bioactivities. A lower FCD value implies higher similarity between two distributions, which in the context of training vs. sampled data implies more effective learning of the training set. The results are collected in Table 1, where each value shown represent the mean ± standard deviation across the 5 cross-validation folds. It can be observed that the SELFIES-based models all have a clear advantage over the corresponding SMILES ones in terms of generation validity due to their full validity. The random placement of the initial token for both bidirectional slightly impacted the model performances in terms of the generation validity and novelty, in agreement with the findings in^7^.

A potential downside of using SELFIES encoding is the increase in the FCD values compared to the SMILES-encoded models. This suggests that the learned distribution diverges somewhat from the original training set embedding manifold. In our opinion, this can be explained by the more rigid syntactic rules of SELFIES used to ensure validity, making it less transparent when converting from the string representation to the actual structure compared to SMILES. In particular, because SELFIES acts as a formal automaton that follows a set of rules to ensure validity^23^, the meaning of the same token can vary (defined by the overloading rules), and sometimes even be removed in the case where the token is ignored due to rule violation. Overloading is the action of assigning multiple meanings to the same token, and in SELFIES overloading occurs whenever a special token (e.g., [Ring] or [Branch]) appears, where the token following the special token is assigned a numerical value according to a predefined table using hexadecimal indexing. It is thus more difficult for the RNN model to learn these implicit (and arbitrary to a degree) syntactic rules. On the contrary, SMILES encoding ensures (1) unique meaning of symbols, (2) relatively simple syntactic rules such as bracket closing and valid valency, which are easier for generative models to capture. By filtering out the invalid generated species, the surviving outputs tend to fall closer to the training manifold. However, we also note that even though a high FCD value from the training set is typically considered as having a lower performance, this is not necessarily the case for constrained generation scenarios. As will be demonstrated in the next section, the target distribution becomes conditional distributions in the presence of the constraint. This reduces the significance of the FCD metric with the original unconditional distribution, and the deviation from the original distribution combined with the full structural validity and high novelty could favor the SELFIES encoding under such tasks.

**Table 1:** Comparison of performance indicators among the tested model structures, where each trained model is tested via a 5-fold cross-validation scheme. Each model is used to sample 100 molecular strings per fold to predict the uniqueness, validity, and novelty. Each model is used to sample 6,000 novel molecular strings for the FCD calculation.

Encoding	Model	Starting Point	# hidden	% Unique ($\uparrow$)	% Valid ($\uparrow$)	% Novel ($\uparrow$)	FCD ($\downarrow$)
SELFIES	Forward	fixed	1024	$100 \pm 0$	$100 \pm 0$	$94 \pm 2$	$3.7 \pm 0.4$
SELFIES	Backward	fixed	1024	$100 \pm 0$	$100 \pm 0$	$94 \pm 1$	$3.7 \pm 0.2$
SELFIES	BIMODAL	fixed	1024	$100 \pm 0$	$100 \pm 0$	$100 \pm 0$	$7.5 \pm 0.4$
SELFIES	FBRNN	fixed	1024	$100 \pm 0$	$100 \pm 0$	$100 \pm 0$	$16.2 \pm 0.4$
SELFIES	BIMODAL	random	1024	$99.8 \pm 0.4$	$99.8 \pm 0.4$	$99.8 \pm 0.4$	$8.7 \pm 0.8$
SELFIES	FBRNN	random	1024	$99.8 \pm 0.4$	$99.4 \pm 0.8$	$99.4 \pm 0.8$	$15.7 \pm 0.4$
SMILES	Forward	fixed	1024	$99.6 \pm 0.5$	$95 \pm 2$	$74 \pm 4$	$1.7 \pm 0.3$
SMILES	Backward	fixed	1024	$100 \pm 0$	$96 \pm 3$	$79 \pm 5$	$1.96 \pm 0.07$
SMILES	BIMODAL	fixed	1024	$99.0 \pm 0.9$	$87 \pm 3$	$85 \pm 3$	$1.8 \pm 0.2$
SMILES	FBRNN	fixed	1024	$99 \pm 1$	$63 \pm 3$	$61 \pm 3$	$3.1 \pm 0.3$
SMILES	BIMODAL	random	1024	$100 \pm 0$	$84 \pm 4$	$84 \pm 4$	$3.7 \pm 0.3$
SMILES	FBRNN	random	1024	$99.8 \pm 0.4$	$53 \pm 2$	$53 \pm 2$	$6.1 \pm 0.5$

### Bidirectional models explore full chemical space for constrained generation

A key motivation for examining bidirectional models is the requirement for flexible molecular design with constrains, such as enforcing the presence of particular pharmacophores or functional groups that are known to be critical for bioactivity in the generated structure. This practice is commonly used in lead optimization for example, to enhance certain desirable properties of the drug while maintaining the overall efficacy. Bidirectional models naturally allow context to flow in both directions, enabling the model to process information about the existing structure while generating the remaining parts of the molecule around it. In contrast, unidirectional models can only place the constraint at either ends of the final structure, leading to artificial limitations.

To quantify the difference between the two generation schemes more rigorously, we first calculate and compare the sizes of chemical space spanned by unidirectional and bidirectional models. Consider an initial constraint string with length *N*, embedded in a molecular string with total length *M*, and the total number of different tokens in the dictionary being *L*. Assume further, for simplicity, that the constraint string is not entirely composed of self-repeating substrings (e.g., it is not of the form ABCABC but can be of the form ABCABCA). Denoting the set of all possible configurations that can be generated by a unidirectional and bidirectional model as $\mathscr {D}_{\textrm{uni}}$ and $\mathscr {D}_{\textrm{bi}}$, one can analytically calculate the sizes of both sets using a counting argument as:

8 $$\begin{aligned} |\mathscr {D}_{\textrm{uni}}|&= L^{M-N} \nonumber \\ |\mathscr {D}_{\textrm{bi}}|&= L^{M-N} (M - N + 1) \nonumber \\&\quad - \sum _{k=2}^{\lfloor M/N \rfloor } \left[ \prod _{l=1}^{k} \frac{1}{k!} (k - 1)(M - kN + l) L^{M - kN} \right] \end{aligned}$$

where the second term in $|\mathscr {D}_{\textrm{bi}}|$ accounts for over-counted instances where the constraint repeats itself in the generated string. For almost all instances, the first term dominates over the second, so the space spanned by a bidirectional model is approximately $(M-N+1)$ times larger than the one spanned by a unidirectional model.

**Fig. 2:**
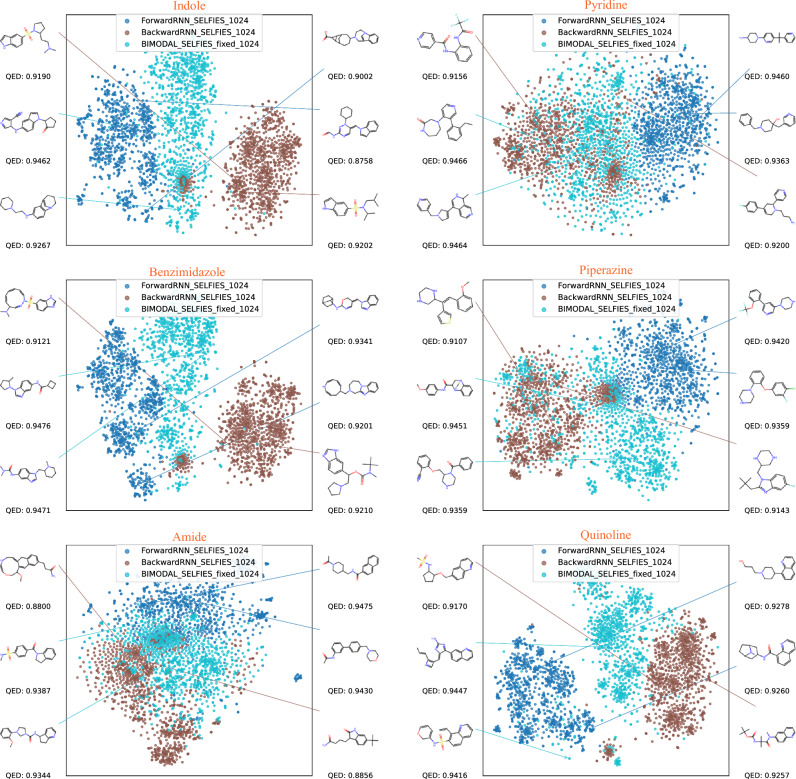
Visualization of space of 1,000 sampled molecules (per model) starting from six different initial SELFIES strings via t-SNE, and structures of top-2-QED molecules from each model.

To further illustrate the importance of bidirectional generation schemes, we designed an experiment to highlight the limitations of relying solely on forward or backward generation. We short-listed the following six different molecular fragments which are commonly seen in bioactive molecules, and used them as starting strings for the different generation models:

1. Indole, SMILES: C1=CC=C2C(=C1)C=CN2, SELFIES: [C][=C][C][=C][C][=Branch1][Ring2][=C][Ring1][=Branch1][N][=C][N][Ring1][=Branch1]
2. Pyridine, SMILES: C1=CC=NC=C1, SELFIES: [C][=C][C][=N][C][=C][Ring1][=Branch1]
3. Benzimidazole, SMILES: C1=CC=C2C(=C1)N=CN2, SELFIES: [C][=C][C][=C][C][=Branch1][Ring2][=C][Ring1][=Branch1][N][=C][N][Ring1][=Branch1]
4. Piperazine, SMILES: C1CNCCN1, SELFIES: [C][C][N][C][C][N][Ring1][=Branch1]
5. Amide group, SMILES: CC(=O)N, SELFIES: [C][C][=Branch1][C][=O][N]
6. Quinoline, SMILES: C1=CC=C2C=CC=NC2=C1, SELFIES: [C][=C][C][=C][C][=C][C][=N][C][Ring1][=Branch1][=C][Ring1][#Branch2]

**Fig. 3:**
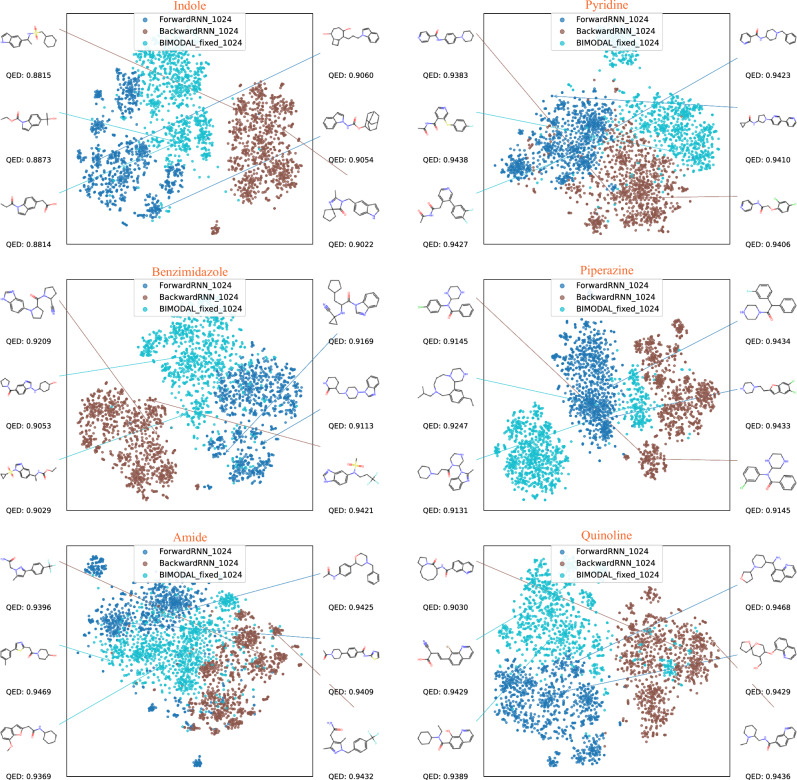
Visualization of space of 1,000 sampled molecules (per model) starting from six different initial SMILES strings via t-SNE, and structures of top-2-QED molecules from each model.

Starting from each SMILES or SELFIES substring, we then used the corresponding (1) Forward model, (2) Backward model, (3) BIMODAL model to sample 200 conditional molecular strings per CV fold, totaling 1,000 molecules. The BIMODAL was selected due to its better performance as demonstrated by our screening results in Table 1. We restrict all produced strings to be unique, valid, and novel. For each generated molecule, we compute the 2048-bit Morgan fingerprint using the GetFingerprint() method from the FingerprintGenerator64 class in RDKit. The Morgan fingerprint is a unique, fixed-length binary vector that captures local atomic environments, and is a commonly used to represent the chemical space structure^46^. The fingerprints are then visualized using a t-distributed stochastic neighbor embedding (t-SNE) algorithm onto a 2-dimensional plane, with perplexity=30. The resulting distributions are shown in Figure 2 for SELFIES strings and Fig. 3 for SMILES strings, respectively. It is clearly visible that for all the initial strings tested, the forward and backward models produce molecules that occupy only part of the constrained chemical space. Moreover, the space occupied by these two models are largely disjoint, with the separation being more significant for larger constraints (Indole and Benzimidazole). This can be understood from Eq.8, since overlapping only occurs when the forward model reproduces the same constraint at the end of the string, and vice versa. A constraint with larger *N* reduces the sizes of unidirectional models, making repeated constraints less likely to occur. Meanwhile, the bidirectional models interpolate between the forward and backward models, filling up the full chemical space. This naturally demonstrates the versatility of bidirectional models, since they naturally include both forward and backward models and everything in between.

When generating with a fixed initial string, the conditional distribution of produced molecules will be different than the unconditional one. Adding a constraint containing bioactive substructures will likely alter the probability of the resulting molecule being a drug. We test this hypothesis by performing Mann-Whitney (MW) and Kolmogorov–Smirnov (KS) tests. The MW test examines whether the median of two sampled sets are different, whereas the KS test examines whether the two sampled sets come from the same distribution. Both tests are non-parametric and applies to our molecular dataset. For both tests, the two sets of samples are (1) the 1,000 constrained molecules, (2) the 30,000 unconstrained molecules from the FCD calculation. The results in Table 2 confirm our hypothesis, as the null hypothesis (distribution unaltered by the constraint) is rejected with at least $97\%$ probability for all cases tested according to the KS test and most have a p-value of 0. One observation from the table is that the backward model always produces more low-QED molecules when conditioning on a structural constraint, compared to generation with a null constraint. We believe this can be attributed to the same “overloading” construction of SELFIES, which we have discussed in Section "Building and Training the Bi-LSTM Models" when comparing the FCD values. Recall that overloading in SELFIES assigns a numerical value to the token following a special token. Such a rule is clearly directional: given the token on the left, it is relatively easy to determine what the next character should be, with access to the assignment table. However, going in the backward direction, it is much harder to decide whether the current token should be interpreted by overloading or not, judging only from the existing tokens on its right. This built-in directionality of SELFIES explains the challenge encountered by the backward model.

Our results suggest that the backward-prediction component could be the limiting factor of bidirectional models, especially when encoded with SELFIES. In particular, this explains the drastic performance difference between BIMODAL and FBRNN models in terms of their FCD value in Table 1, since the former utilizes both forward and backward information when predicting in either direction, while the latter only utilizes backward direction when predicting backwards. Interestingly, the Backward model performs better than FBRNN using SELFIES encoding, implying that sequential predictions using different information from two directions could be detrimental to the overall generation quality.

**Table 2:** Statistical test results regarding QED score distributions between molecules sampled by constrained and non-constrained models. p-values less than $10^{-10}$ are displayed as 0.

Seed	Encoding	Model	Constrained Median	Null Median	Cons. Higher?	MW p-value	KS p-value
Indole	SMILES	Forward	0.5849	0.5217	Y	0	0
Indole	SMILES	Backward	0.5505	0.5217	Y	1E-6	3E-7
Indole	SMILES	BIMODAL	0.4697	0.5150	N	2E-10	7E-10
Indole	SELFIES	Forward	0.4897	0.5201	N	4E-6	0
Indole	SELFIES	Backward	0.3606	0.5312	N	0	0
Indole	SELFIES	BIMODAL	0.5407	0.5189	Y	3E-3	8E-4
Pyridine	SMILES	Forward	0.6750	0.5217	Y	0	0
Pyridine	SMILES	Backward	0.5424	0.5212	Y	2E-3	1E-2
Pyridine	SMILES	BIMODAL	0.5344	0.5150	Y	2E-3	3E-2
Pyridine	SELFIES	Forward	0.5642	0.5201	Y	0	0
Pyridine	SELFIES	Backward	0.3934	0.5312	N	0	0
Pyridine	SELFIES	BIMODAL	0.5818	0.5189	Y	0	2E-10
Benzimidazole	SMILES	Forward	0.6054	0.5217	Y	0	0
Benzimidazole	SMILES	Backward	0.6027	0.5211	Y	0	0
Benzimidazole	SMILES	BIMODAL	0.4840	0.5150	N	9E-6	2E-7
Benzimidazole	SELFIES	Forward	0.5500	0.5200	Y	3E-6	0
Benzimidazole	SELFIES	Backward	0.3703	0.5312	N	0	0
Benzimidazole	SELFIES	BIMODAL	0.5610	0.5189	Y	2E-7	3E-6
Piperazine	SMILES	Forward	0.6306	0.5217	Y	0	0
Piperazine	SMILES	Backward	0.7514	0.5212	Y	0	0
Piperazine	SMILES	BIMODAL	0.5832	0.5150	Y	0	0
Piperazine	SELFIES	Forward	0.6052	0.5201	Y	0	0
Piperazine	SELFIES	Backward	0.3600	0.5312	N	0	0
Piperazine	SELFIES	BIMODAL	0.5479	0.5189	Y	1E-3	1E-3
Amide	SMILES	Forward	0.5883	0.5217	Y	0	0
Amide	SMILES	Backward	0.4386	0.5212	N	0	0
Amide	SMILES	BIMODAL	0.4497	0.5150	N	0	0
Amide	SELFIES	Forward	0.6167	0.5201	Y	0	0
Amide	SELFIES	Backward	0.2612	0.5312	N	0	0
Amide	SELFIES	BIMODAL	0.4138	0.5189	N	0	0
Quinoline	SMILES	Forward	0.6148	0.5217	Y	0	0
Quinoline	SMILES	Backward	0.5271	0.5212	Y	0.86	5E-3
Quinoline	SMILES	BIMODAL	0.5906	0.5150	N	0	0
Quinoline	SELFIES	Forward	0.5181	0.5201	N	0.90	4E-5
Quinoline	SELFIES	Backward	0.4370	0.5312	N	0	0
Quinoline	SELFIES	BIMODAL	0.5524	0.5189	Y	5E-4	3E-5

**Fig. 4:**
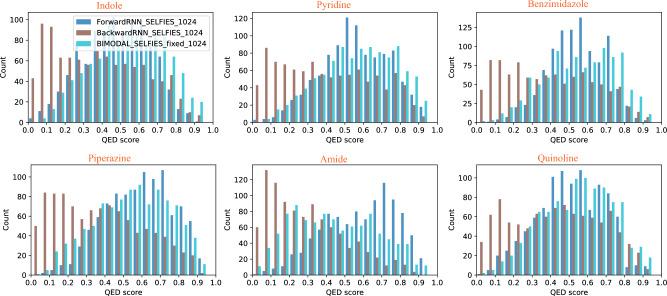
QED score distribution of 1,000 generated molecules (per model) starting from six different initial SELFIES strings.

**Fig. 5:**
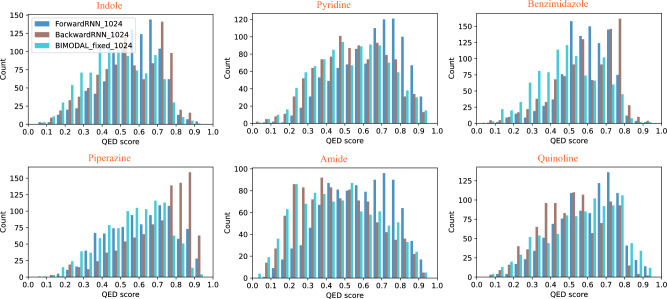
QED score distribution of 1,000 generated molecules (per model) starting from six different initial SMILES strings.

The behavior of bidirectional vs. unidirectional models is further confirmed by the distribution of Quantitative Estimate of Drug-likeness (QED) scores, shown in Fig. 4 for SELFIES and Fig. 5 for SMILES. The distribution of molecules produced by the bidirectional model is more evenly distributed across the entire spectrum of QED, and in particular, is interpolating those from the two unidirectional models. We observe that for SELFIES-based generation, the BIMODAL model tends to produce the highest-QED molecules, whereas for SMILES-based generation, there is no clear trend on which model produces the highest-QED molecules consistently. It is also observable from the plots how the Backward SELFIES models are underperforming compared to the two other models. Finally, visualizations of the molecules with top QED scores in Figs. 2 and 3 confirm that the produced molecules all retain the target constraint structure, and both SELFIES and SMILES encoding are capable of producing highly drug-like molecules, despite the increased FCD values of the SELFIES-based models.

Importantly, the form of the initial string determines the attachment point for the substructure, which is typically the atom at both ends of the constraint string by the syntactic rules of SMILES and SELFIES. This behavior has been acknowledged in the literature^9^ and here we elaborate a little more. Take the first structure, indole, as an example. Using the SMILES string: C1=CC=C2C(=C1)C=CN2 as the starting point of generation, the additional structures produced by the RNN model will attach to the initial carbon atom and the final nitrogen atom. The same rule applies similarly in the SELFIES representation. By making use of the non-uniqueness of both encoding rules, generated structures can also be attached to different locations of the constraint substructure. By the same reasoning, an important rule when constructing the initial strings is to always maintain an open valence for both attaching atoms. Failures to do so will result in either (1) an invalid SMILES string, or (2) generated SELFIES strings being omitted due to valence rule violation. An example of invalid initial string for the same indole molecule would be: C1=CC=C2C(=C1)CC=N2, where the last nitrogen atom forms 3 bonds with neighboring carbons and thus has no open valency. Further strings produced from this substring in the forward direction will always lead to incorrect SMILES structures .

### Model fine-tuning for desired properties

The next main component of BiRLNN is the utilization of RL to guide the pre-trained Bi-LSTM model to produce highly-desired molecules with higher probability. In practical molecular design, one often faces the challenge of optimizing a multi-objective function^47^. Here, we choose to simultaneously optimize the candidate molecules such that they have both a high QED score, implying a higher likelihood that they are potential drugs, and a low SA score, meaning that they are easy to synthesize. This can be achieved by adjusting the weights in the reward function, Eq.6, where $w_q$ for the QED score would be positive while $w_s$ for the SA score would be negative.

We demonstrate the RL fine-tuning using the FBRNN-SELFIES model. Starting from a randomly selected pre-trained model from fold-2, epoch-10, we pass it to an in-house REINFORCE module where at each episode, a batch of $K=32$ molecules are generated starting from a null initial string with a temperature parameter $T=1.0$, and their reward values are used to update the parameters in FBRNN according to Eq.7. The optimization is done using an AdamW optimizer in torch.optim with a learning rate of $10^{-4}$ and a weight decay of $10^{-2}$. The entropy coefficient $\beta$ in Eq.7 is set to $10^{-4}$. Starting from each of the six initial constrained substring, we conduct the RL optimization for two different sets of weights $(w_q, w_s)$ (Eq.6) where $w_q$ is the QED weight and $w_s$ is the SA weight: $(w_q, w_s)=(1,0)$ or (1, 1). Correspondingly, the target functions are

9 $$\begin{aligned} p_q = \text {QED},\ p_s = -\frac{(\text {SA}-1)}{9} \end{aligned}$$

where $p_q$ represents the raw QED score of generated molecules, and $p_s$ represents the rescaled negative SA score which is between $-1$ and 0. The rescaling maps the SA score to be on the same scale as the QED score, and the negative sign implies that we want lower SA scores. In each case, the model is trained for a total of 120 episodes. We record the mean and standard deviation of the QED score and SA score at the beginning and end of the training, in Table 3. For the weight (1, 0) case, only the QED score enters the reward function and is being optimized. For all initial strings, there is a significant increase in the mean QED score at the end of fine-tuning compared to the beginning. Interestingly, we also observe a relatively small decrease in the SA score for all seeds except Quinoline whose SA mean remains unchanged. This is likely due to the partial overlap between desirability functions of the QED score, with the inverse of SA’s penalty components. Since factors contributing to a high QED include intermediate molecular weight and low rotatability, these can partially align with conditions for a low SA. For the weight (1, 1) case, both QED and SA are being actively optimized. For all initial seeds there is a significant increase in the mean QED score, and a significant decrease in the mean SA score. At the same time both QED and SA show a decrease in the standard deviation to about 50 to $70\%$ the original value at the end of the training compared to the beginning, except for the Amide group which shows no decrease in the SA standard deviation. This indicates that the agent is slowly converging to a fewer superior solutions but not yet fully collapsed onto a small subset. We attribute this to the low learning rate, which is effective in delaying the mode collapse and allows the model to explore more regions of the chemical space.

**Fig. 6:**
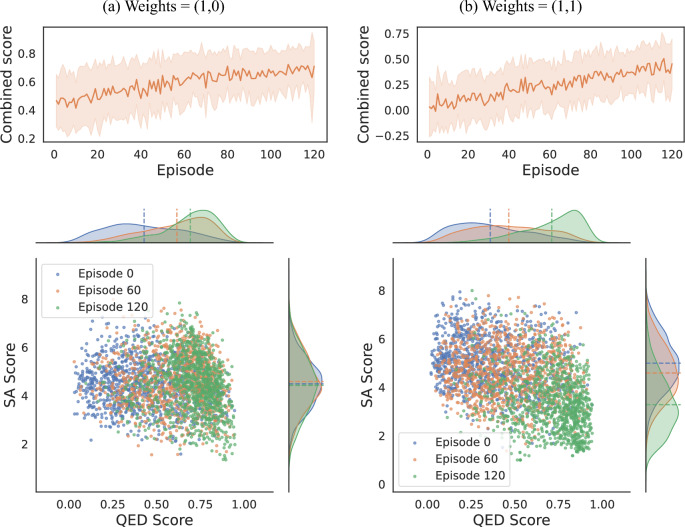
Reinforcement learning results using FBRNN-SELFIES model with Amide group as starting substring. Top: Moving average of the reward function over 120 episodes of RL training. Bottom: QED and SAScore distributions for 1,000 molecules generated using models after different numbers of episodes of RL training. Left: weights for QED ($w_q$) and SA ($w_s$) are $(w_q, w_s)=(1,0)$. Right: weights are $(w_q, w_s)=(1,1)$.

We visualize the RL training process in Fig. 6 using Amide as an example. For each weight combination, we plot the batch-averaged final reward as a function of episode number, along with the sample standard deviation of the rewards. Then, we use each of the RL-trained agents saved at different episode numbers to sample 1,000 molecules, and compute the two quantities we optimized over. The left and right panels show the case where weights are (1, 0) and (1, 1). The training reward curves on the top shows a steady increase in the average reward per episode, indicating effective learning by the agent. The plots on the bottom show the distribution of molecules produced by different models as the training proceeds. For both weight pairs, an increase in the median QED score indicated by the vertical dotted line is clearly visible for models trained with more episodes. Meanwhile, there is only a small change in the median SA score for weight (1, 0), but a much larger change for weight (1, 1) where SA is being actively optimized. This confirms the success in RL training, as both the optimization targets have moved towards the desired direction. Overall, the resulting broad distributions for both weight pairs show that up to episode 120, the model is still sampling highly diversely within the chemical space while acquiring a higher probability of producing desirable products.

**Table 3:** RL training statistics at beginning and end of RL trianing for FBRNN-SELFIES model, constrained on different initial strings and for different optimization weights. The values correspond to mean and standard deviations of 32 samples generated with temperature 1.0 during each training episode.

Seed	Weight	Episode	QED Mean	QED Std. Dev.	SA Mean	SA Std. Dev.
Indole	(1,0)	0	0.5774	0.1903	3.8110	0.9782
Indole	(1,0)	120	0.7110	0.1388	3.3201	0.8625
Indole	(1,1)	0	0.5811	0.1929	3.7925	1.0070
Indole	(1,1)	120	0.7196	0.1148	2.6364	0.4334
Pyridine	(1,0)	0	0.4976	0.2180	4.2320	1.0206
Pyridine	(1,0)	120	0.6836	0.1474	3.2736	0.9652
Pyridine	(1,1)	0	0.4781	0.2126	4.1915	0.9690
Pyridine	(1,1)	120	0.7137	0.1081	2.5699	0.6755
Benzimidazole	(1,0)	0	0.5588	0.1928	3.9483	1.0613
Benzimidazole	(1,0)	120	0.6922	0.1226	3.2594	0.8061
Benzimidazole	(1,1)	0	0.5570	0.1957	3.9194	0.9829
Benzimidazole	(1,1)	120	0.6947	0.1098	2.7966	0.5749
Piperazine	(1,0)	0	0.4341	0.2061	4.9633	0.9272
Piperazine	(1,0)	120	0.7129	0.1450	3.8751	0.9612
Piperazine	(1,1)	0	0.4403	0.2066	5.0388	0.9862
Piperazine	(1,1)	120	0.7401	0.1055	3.3219	0.5605
Amide	(1,0)	0	0.4290	0.2097	4.4902	0.9986
Amide	(1,0)	120	0.6928	0.1506	4.4257	1.1810
Amide	(1,1)	0	0.4433	0.2101	4.4962	1.0053
Amide	(1,1)	120	0.7110	0.1683	3.2781	1.1776
Quinoline	(1,0)	0	0.5509	0.2052	4.2232	1.0136
Quinoline	(1,0)	120	0.7576	0.1209	3.8904	1.0769
Quinoline	(1,1)	0	0.5549	0.2047	4.2507	1.0560
Quinoline	(1,1)	120	0.7693	0.0997	2.4271	0.7493

**Fig. 7:**
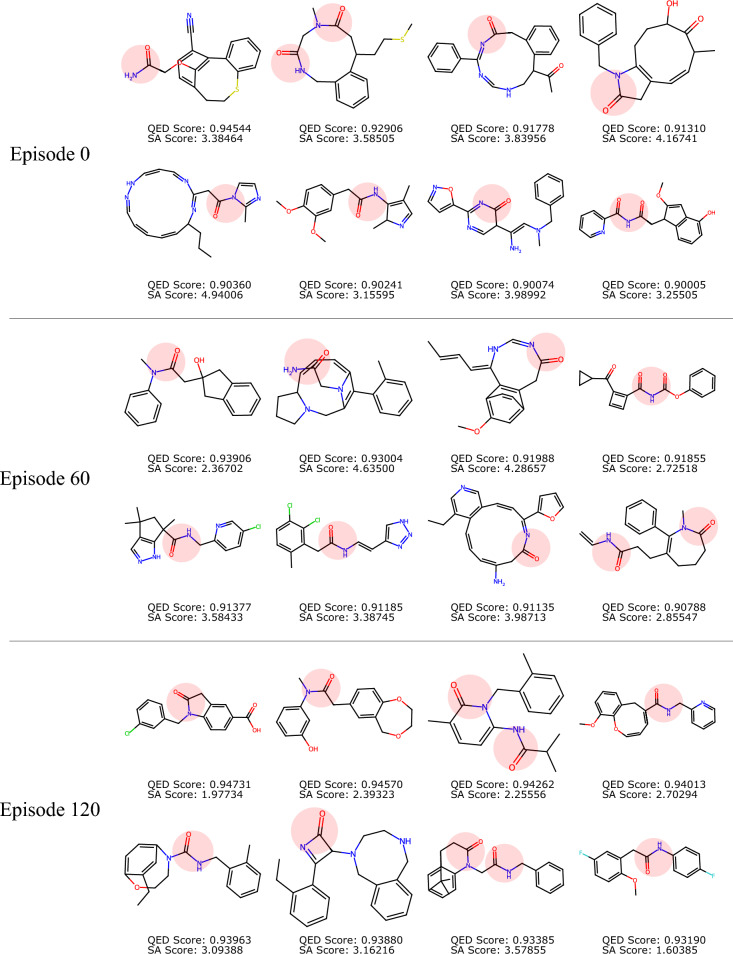
Selected top molecules generated by the model with different RL training episodes, for $(w_q, w_s)=(1,0)$. Constraint structure highlighted in red.

**Fig. 8:**
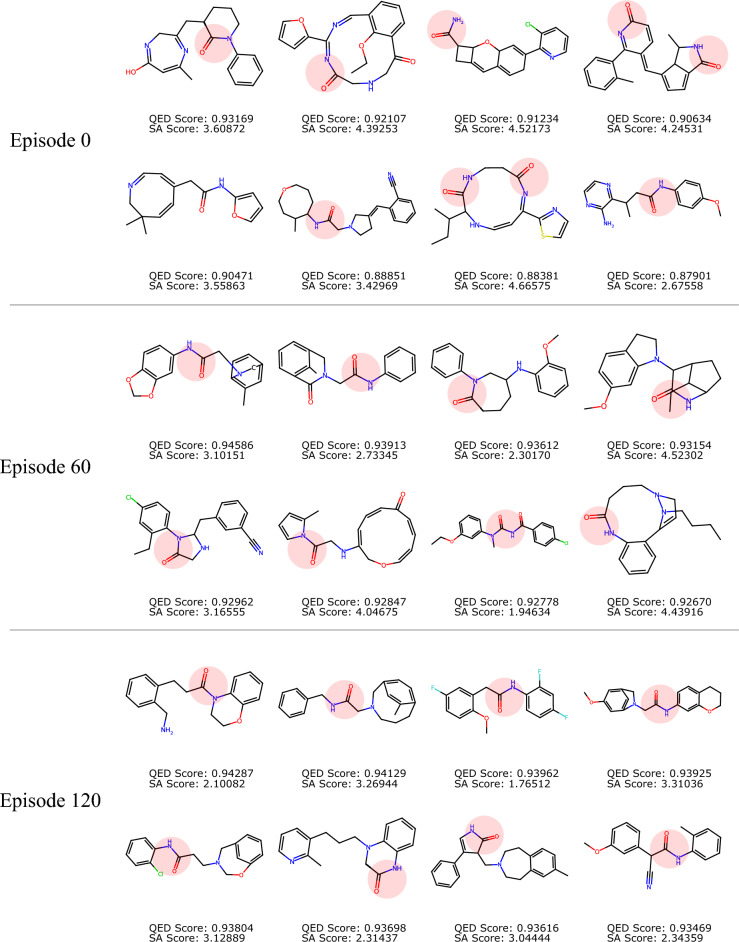
Selected top molecules generated by the model with different RL training episodes, for $(w_q, w_s)=(1,1)$. Constraint structure highlighted in red.

As the goal of de novo drug discovery tasks typically require finding only a small subset of high-quality molecules, instead of a large set of suboptimal ones, it is helpful to also evaluate the efficacy of a particular model by examining the best molecules it can produce. Figures 7 and 8 show the unique molecules with top QED scores among the 1000 generated ones, sampled by the FBRNN model at different RL episodes with training weights (1, 0) and (1, 1), respectively. It can be observed that our conclusion based on collective statistics of the generated molecules applies similarly to the top molecules. For the case with weight (1, 0), the QED scores of the top molecules increases with the training episodes, while there is no observable change in the SA score distribution. The resulting structures at episode 120 contains large, multi-heteroatom rings and have relatively high SA scores. For the case with weight (1, 1), the generated molecules achieve both a high QED score and a low SA score simultaneously at high training episodes. The resulting structures at episode 120 contains a smaller fraction with large rings, while other high-QED molecules contain simpler ring and linker structures.

We point out that due to the stochastic nature of deep learning models, molecules generated by them can exhibit high structural variations. In some circumstances, such models can produce chemically unrealistic molecules, which are either unlikely to exist or exhibit high instability. This chemical implausibility could be reduced via usage of filters such as SA score, at the expense of a higher computational cost, yet will be difficult to eliminate fully. Specifically, it can be observed from Figs. 7 and 8 that some high-reward molecules still include large or highly substituted macrocycles, which are known to be challenging to synthesize in reality. This observation also highlights a well-recognized limitation of heuristic metrics such as the SA score, rather than a flaw in the reinforcement learning algorithm itself. The tendency of RL agents to exploit imperfections in proxy reward functions is common in molecular optimization frameworks^14,48,49^. In our context, BiRLNN correctly identifies regions of chemical space that minimize the SA function as defined, even if these do not always align with human-intuitive synthetic feasibility. Practically, a more realistic synthesizability assessment may be complemented with retrosynthesis-based analysis using e.g. AiZynthFinder^50^, which quantifies the number of valid synthetic routes and the corresponding route lengths. Such multi-metric analysis ensures that the reported improvements in the reward are chemically interpretable and not artifacts of a single heuristic metric.

## Discussion

In this paper, we presented the BiRLNN framework for molecular design, demonstrating its efficacy in generating novel molecular structures with desired properties. Our method combines the power of bidirectional LSTM networks with robust SELFIES encoding, capturing the full chemical space in a constrained molecular design task. By visualizing the chemical spaces using molecular fingerprinting, we illustrated the importance of utilizing bidirectional schemes over unidirectional ones in order to explore all possible products. Moreover, we demonstrated that by constructing a multi-objective reward function, the BiRLNN framework can adaptively bias generation toward promising regions of chemical space, leading to the discovery of high-quality molecules with desired pharmacological profiles.

Looking ahead, it will be highly desirable to extend the capability of BiRLNN by incorporating more advanced rewards into the RL optimization target. These can include not only conventional ADMET (absorption, distribution, metabolism, excretion, and toxicity) properties, but also factors such as blood-brain barrier permeability or binding affinity to particular target proteins. Such rewards may need to be computed either via deep learning methods, quantum computing/simulation methods, or a combination of both. On the one hand, many prior works have demonstrated the effectiveness of using deep learning models as surrogate predictors for guiding molecular optimization. For example, pre-trained graph neural networks (GNNs) have been employed to estimate complex properties such as blood-brain barrier permeability^51^, toxicity^52^, and protein-ligand binding affinity^53,54^. Similarly, models such as DeepTox^52^ or ToxinPredictor^15^ can provide toxicity estimation on generated molecules, which can increase the safety of generated drug candidates. On the other hand, quantum computing may offer an alternative route to more efficiently computing some of the rewards, due to the quantum mechanical nature of molecules. Advanced quantum algorithms such as quantum phase estimation^55,56^ or quantum signal processing^57,58^ can be invoked to perform Hamiltonian simulation, providing key characteristics of the candidates including ground/excited state energies and reaction pathways.

Next, it is beneficial to improve the simple REINFORCE algorithm in BiRLNN to achieve more robust target optimization. Note that RL-based molecular generation inevitably involves a trade-off between reward maximization and chemical diversity. When over-trained, the policy will repeatedly exploit a limited set of high-reward solutions, leading to diminished structural variety and limited exploration of alternative possibilities. In practice, several strategies can mitigate this issue. First, early stopping during fine-tuning can preserve diversity by halting training before the model overfits to the reward landscape. Second, incorporating entropy bonuses into the policy objective encourages exploration by penalizing overly deterministic action distributions, thereby maintaining stochasticity in token generation. Third, the inclusion of diversity-promoting regularizers can help balance property optimization with exploration of new chemical regions. Examples include penalties for generating duplicate scaffolds, or rewards proportional to molecular novelty. In future extensions of BiRLNN, we plan to combine these techniques with adaptive reward scaling or multi-objective optimization (e.g., Pareto-guided reinforcement learning) will further stabilize training and yield models that produce molecules that are both high-performing and chemically diverse.

Another challenge lies in how different target functions can be integrated together in the reward, especially when some functions can be mutually contradicting. One needs to prevent the model from collapsing to sub-optimal solutions defined by the reward function, which can lead to undesired targets since it is challenging to construct the exact optimal reward prior to clinical experiments^27^. As the landscape of molecular design evolves, we anticipate the need for dynamic reward functions that can adapt to emerging targets and design goals. A more advanced exploration strategies could also allow the model to avoid premature convergence and explore a broader chemical space. Moreover, since both deep learning or quantum computing estimators could be expensive to invoke, an important task is to reduce the number of times such oracles are invoked, in order to speed up the optimization process. Notable recent efforts to improve efficiency include using data augmentation and/or experience replay^33,59^, or using active learning to approximate expensive oracle functions^34^. It would also be interesting to explore whether additional RL algorithms, such as PPO studied in a recent work^39^, can offer additional advantages in balancing exploration and exploitation for our framework.

Finally, a more systematic initial string preparation would greatly benefit the constrained generation task, for both SMILES and SELFIES encodings. On the one hand, as mentioned in Section "Bidirectional models explore full chemical space for constrained generation", the non-uniqueness of both encoding schemes implies that one substructure can have multiple string representations, and each would correspond to the additional structures being attached differently on top of the substructure. Therefore, to fully realize the potential of one substring, we need to construct all variations when encoding the same structure, and combine the strings generated from all possible encodings. On the other hand, while we have manually selected the initial strings in our experiments, a natural question is how can such initial constraints be automatically determined for a particular task. Previous works have established that similarity between SELFIES strings does not reliably reflect molecular similarity^19,60^, due to the non-uniqueness and syntactic variability of valid string encodings for the same structure. Therefore, clustering based on string-level distances often fails to group chemically related compounds in a meaningful way. A more robust strategy could be to cluster molecules using chemically informed representations, such as molecular fingerprints^61^, scaffold-based decompositions^9,62^, or learned graph embeddings^63^, so that shared structural motifs can be extracted as candidate constraints for bidirectional generation. This could enable more automated, task-specific initialization schemes while preserving chemical relevance.

## Data Availability

The code used in this work can be found at: https://github.com/nrc-cnrc/BiRLNN. The training datasets and produced results can be found at: https://zenodo.org/records/17059868.
